# Internal carotid artery pseudoaneurysm after neck manipulation in a patient with Eagle syndrome

**DOI:** 10.1002/ccr3.8814

**Published:** 2024-04-29

**Authors:** Ahmed Gamal Sayed, Hesham Aboloyoun, Yasmine Saad Makarem, Ashraf Elnaggar

**Affiliations:** ^1^ College of medicine, Alfaisal University Riyadh Saudi Arabia; ^2^ Vascular and Endovascular Surgery Department Faculty of Medicine, Assiut University Assiut Egypt; ^3^ Rheumatology and Rehabilitation Department Faculty of Medicine, Assiut University Assiut Egypt

**Keywords:** cerebrovascular stroke, chiropractic, Eagle syndrome, endograft, internal carotid artery, pseudoaneurysm

## Abstract

**Key Clinical Message:**

Contraindications of spinal manipulative therapy (SMT) have been proposed, which mandate rigorous control for its safe practice when manipulating the upper spine. Here, we report a vascular complication of Neck Manipulation in Eagle syndrome (ES) patient.

**Abstract:**

SMT is used to treat musculoskeletal conditions such as back pain and neck pain by applying force to the spinal joints. Here, we report a rare but devastating complication of SMT, where a young male patient, 22 years old, with ES, had a large pseudoaneurysm from the internal carotid artery (ICA) after SMT from an unlicensed practitioner, treated successfully with endograft. Clinicians administering SMT should consider an elongated styloid process as a potential contraindication to this therapy.

## 1 INTRODUCTION

SMT entails a range of manual maneuvers that stretch, mobilize, or manipulate the spine, paravertebral tissues, and other joints to relieve spinal pain.^1^ Manipulation of the spine differs from mobilization, as it involves a sudden applied thrust. In contrast, mobilization involves a low‐velocity, passive movement that can be limited or even halted by the patient.^2^ Numerous absolute and relative contraindications of SMT have been proposed.^3^, ^4^, ^5^ The safety of SMT requires rigorous control.

Spinal manipulation rarely causes severe adverse events such as rib fractures and has rarely been associated with neurological and vascular sequelae.^6^ The reported cerebrovascular insults were primarily due to vertebral artery dissection.^7^ Injuries of the cervical internal carotid artery (ICA) are less often associated with SMT, probably because it lies in the soft tissue of the neck and is thus more mobile. The ICA has seven segments: cervical, petrous, lacerum, cavernous, clinoid, ophthalmic, and communication.^8^ The cervical segment begins at the level of C3 and ends at the skull base, usually having no branches.^9^ Flexion‐extension trauma is more likely to injure the carotid arteries, whereas rotational trauma more often damages the vertebral arteries.^10^, ^11^

Eagle syndrome (ES) is a rare clinical syndrome characterized by elongating the styloid process or calcifying the stylohyoid ligament. ES is a risk factor for carotid artery injury, and prior ICA pseudoaneurysm has been reported in this syndrome in the absence of SMT.^11^ ES is a complex symptom assortment produced by provocation of the carotid space structures by anomalies of the styloid process,^12^ including an elongated styloid of 30 mm or larger,^13^ insulting angulation, calcification of the stylohyoid or stylomandibular ligaments, and/or approximation of the styloid to C1 transverse process, commonly seen with a styloid of normal length.^14^

## 2 CASE REPORT

### 2.1 History and examination

A 22‐year‐old male patient presented with a history of neck pain for 10 days, for which he had muscle relaxants by himself and SMT that was done by a non‐licensed practitioner. This was followed immediately by exacerbation of neck pain and swelling.

### 2.2 Differential diagnosis, investigations, and treatment

**FIGURE 1:**
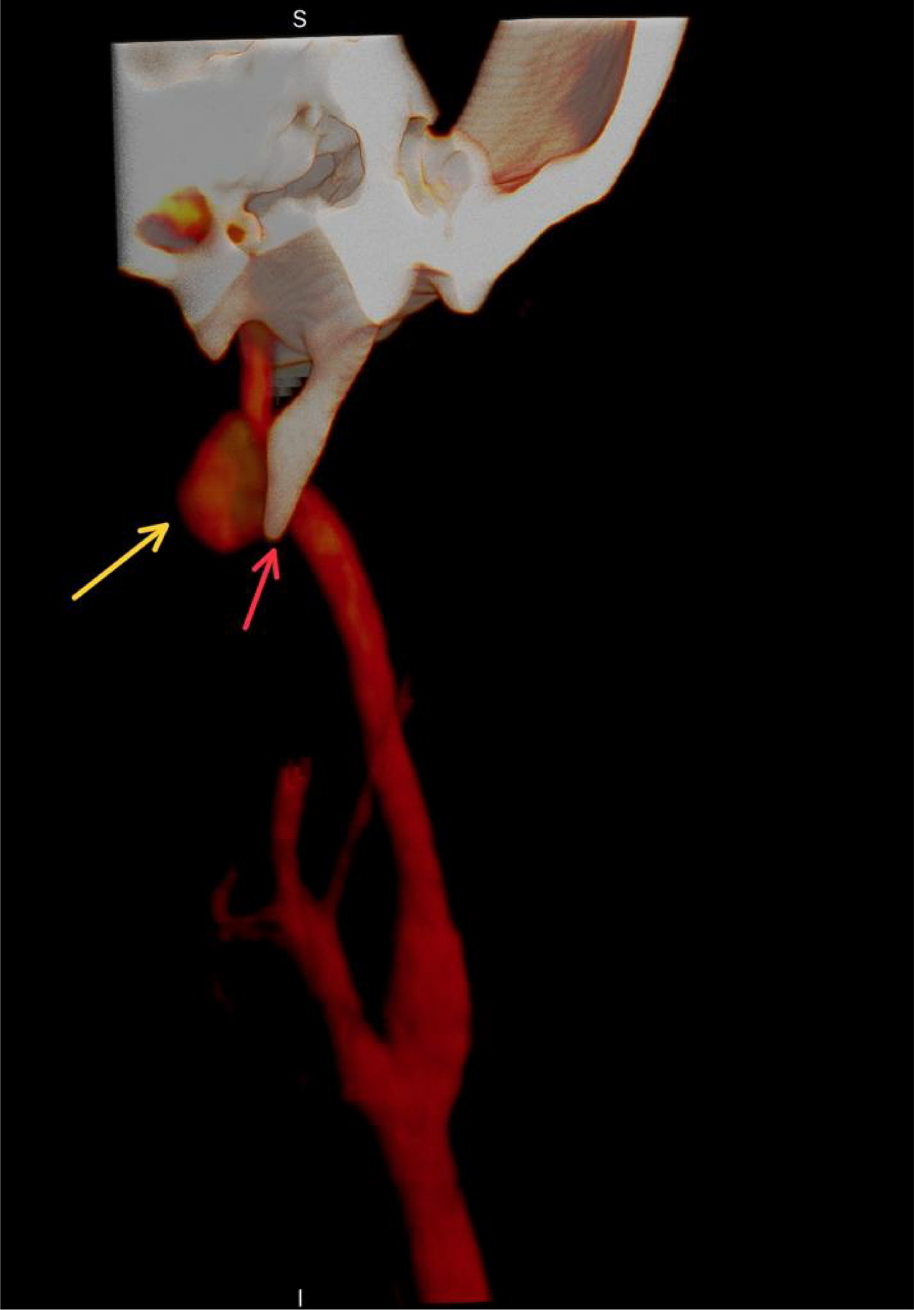
The yellow arrow points to the pseudo‐aneurysm, and the red arrow points to the styloid process.

One week later, he had torticollis and recurrent bleeding attacks from nose and mouth, after which he sought otorhinolaryngologist advice, who requested CT angiography, which revealed an elongated styloid process alongside a nasopharyngeal hematoma and pseudo‐aneurysm from the distal cervical segment of the left ICA just below the skull base (Figure 1). The patient was anemic but hemodynamically stable.

The right common femoral artery (CFA) was percutaneously accessed under local anesthesia with (5 mL xylocaine 2%), and the anesthesiologist continuously monitored the patient's vital signs. Flexor shuttle sheath (COOK® 6Fr. 90 cm) was navigated over 0.035‐in. diameter soft glide wire (Terumo, Tokyo, Japan) together with bern (Cook, Inc., Bloomington, IN, USA) catheter till the left common carotid artery (CCA).

**FIGURE 2:**
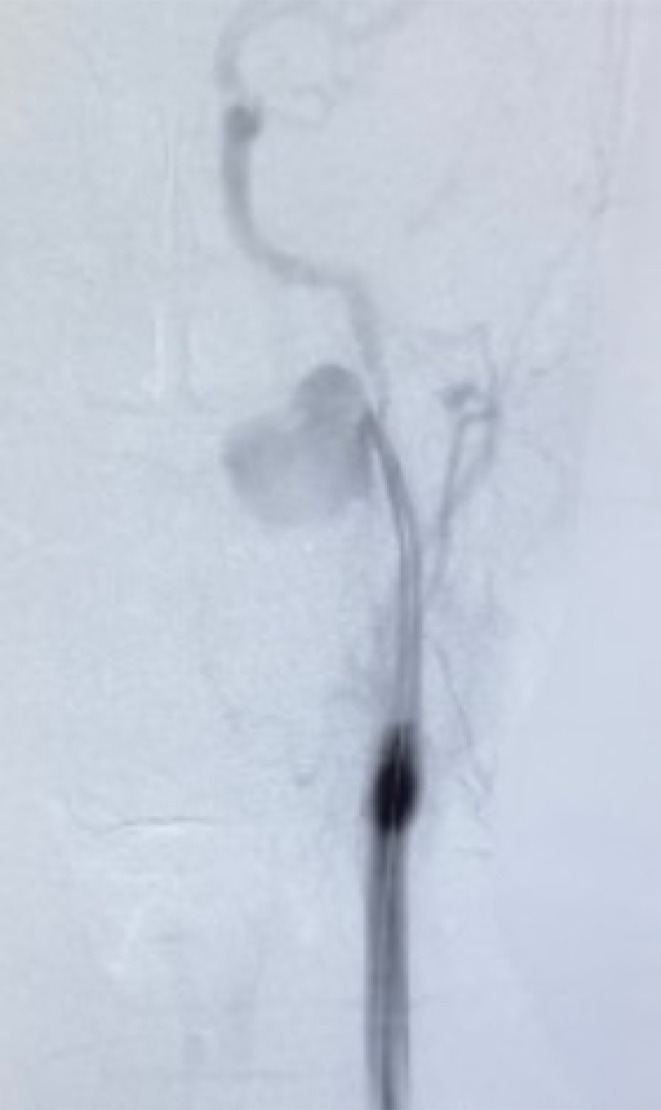
Diagnostic angiography confirming the site of the tear in the left ICA.

**FIGURE 3:**
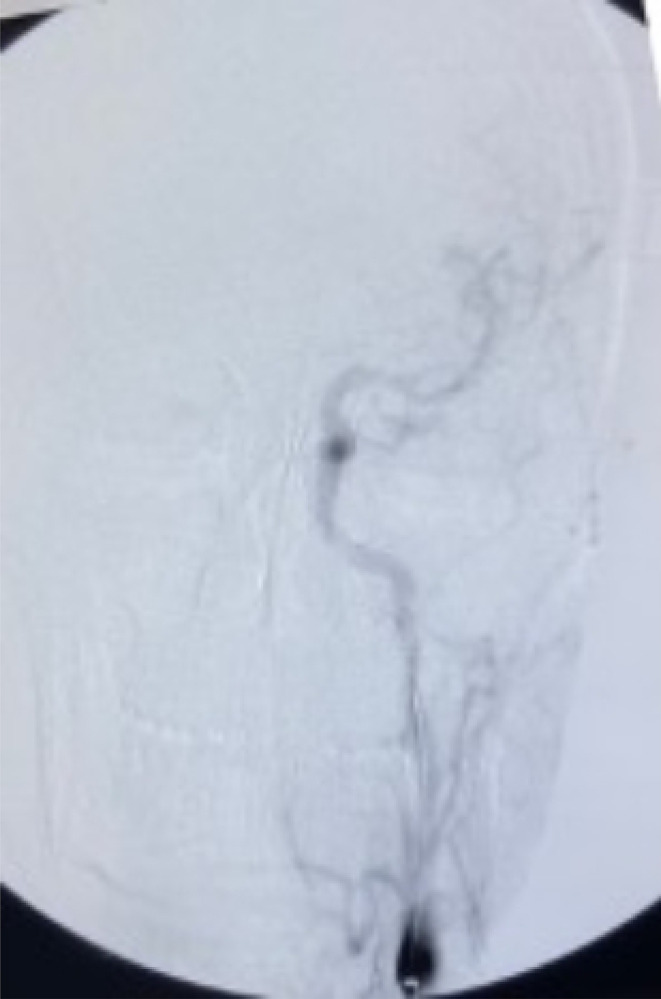
Completion angiography confirming good sealing of the tear and restoration of antegrade flow to the brain.

Diagnostic angiography with manual hand injection of 10 mL of iopromide (Ultravist®) over 5 sec confirmed the site of injury (Figure 2). BeGraft (Bentley InnoMed GmbH, Germany) (6mm × 38mm) covered stent graft was then advanced till the end of the straight part of the cervical segment of ICA; guided by the help of roadmap and deployed via inflating of its balloon with 8 atm pr. for 30 sec. Nitroglycerine was then injected, and completion angiography revealed good sealing of the tear and restoration of the antegrade flow to the brain (Figure 3).

### 2.3 Outcome and follow‐up

**FIGURE 4:**
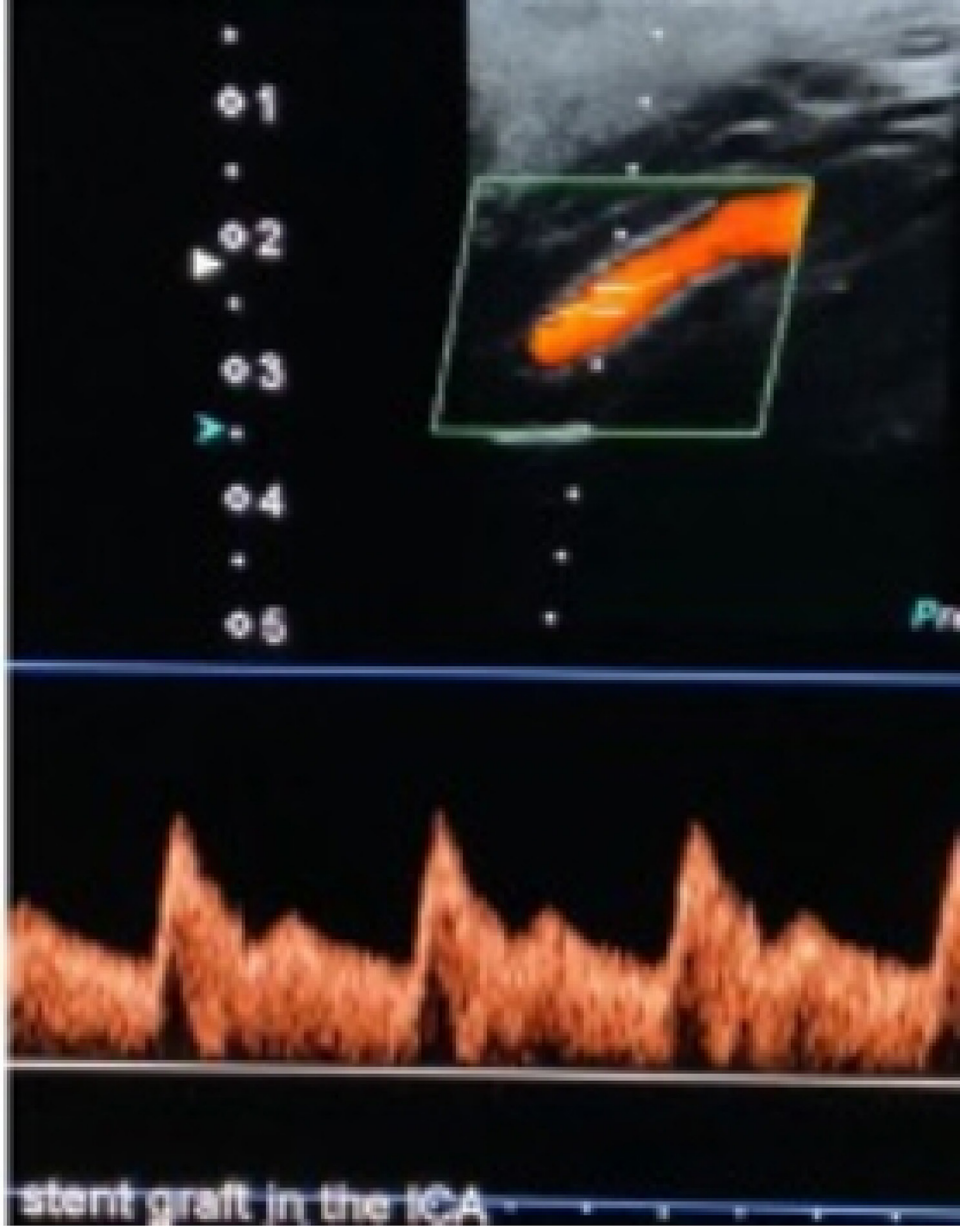
Follow‐up duplex showing patent stent with normal ICA waveform.

**FIGURE 5:**
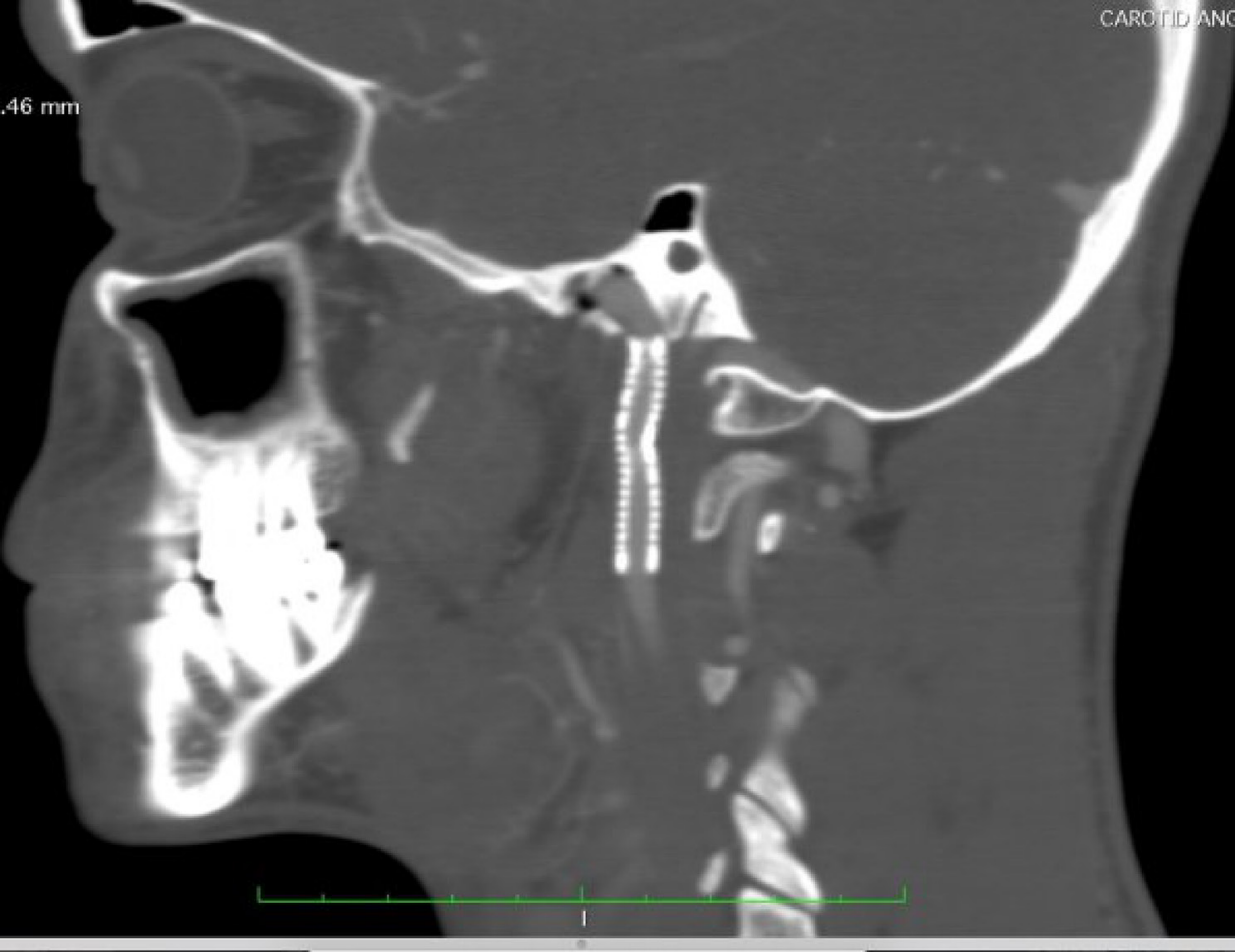
One‐month postoperative CTA revealing patent stent‐graft.

The patient was discharged on broad‐spectrum antibiotics, clopidogrel, and low molecular weight heparin (LMWH). After 3 days, the last one was discontinued to be continued on clopidogrel. At 30 days postoperatively, a duplex ultrasound showed a patent stent graft with normal ICA‐waveform (Figure 4). CTA (Figure 5) confirmed that the patient's neck pain was resolved and stopped experiencing spitting blood anymore.

## 3 DISCUSSION

ES, categorized initially by Dr. Watt Eagle, into two groups based on the structures compressed or irritated by the styloid complex. The classic form involves cranial nerves 5‐trigeminal, 7‐facial, 9‐glossopharyngeal, and/or 10‐vagus, where many providers believe this neuralgia is a type of entrapment syndrome involving the cranial nerves, commonly after tonsillectomy.^15^ The vascular form involves the ICA, external carotid artery (ECA),^12^ periarterial sympathetic nerve plexus,^16^ and the internal jugular vein (IJV).^17^, ^18^

In our patient, we assume that the muscle relaxant weakened the normal muscle tone and weakened its share in neck support. On the other hand, SMT even in the hands of experienced practitioners, has well‐known complications, particularly for the upper cervical segment. The pathophysiology of such complication is the presence of an anomalous styloid process that caused the injury to the ICA. SMT may have acted to further press the anomalous styloid against the ICA or stretch the artery against this bony process, causing injury.

Many patients who unknowingly have ES pursue physical therapy, massage, medical management, injections, and surgery.^19^ Clinicians who use SMT should be aware of the signs and symptoms of cervical vascular pathology and be able to identify and refer such patients for appropriate medical care.^20^ However, three studies described carotid artery dissection and/or stroke after massage.^21^, ^22^, ^23^ Another three studies described exercise produced adverse symptoms/events, including arterial dissection.^24^, ^26^, ^29^ Another research recommends the avoidance of thrust manipulation, along with relative contraindications for combined flexion/rotation in patients with styloid anomalies, as this has led to carotid dissection in several cases.^25^, ^27^ Due to the relative inaccessibility of the distal ICA, we chose the endovascular choice as the endovascular management of carotid artery injury that had been previously studied and proved to be safe, effective, and maybe even superior to surgery, particularly in zone I and III neck injuries.^28^, ^29^, ^30^, ^31^

## 4 CONCLUSIONS

This case highlights a 22‐year‐old man who suffered a bleeding ICA pseudoaneurysm following SMT, which was managed by stent grafting with good recovery. Practitioners of SMT should consider an elongated styloid process or ES as a potential contraindication to cervical spine SMT due to the proximity of the abnormal styloid to the ICA. Patients undergoing spinal manipulative therapy (SMT) should be informed of the risk of stroke or vascular injury that could cause significant morbidity or even mortality from this procedure. Furthermore, we recommend increasing the public's awareness about the potential complications of SMT. In particular by non‐licensed providers.

## AUTHOR CONTRIBUTIONS

**Ahmed Gamal Sayed:** Project administration; software; validation; writing – review and editing. **Hesham Aboloyoun:** Validation; writing – review and editing. **Yasmine Saad Makarem:** Data curation; writing – review and editing. **Ashraf Elnaggar:** Supervision; validation; writing – original draft.

## FUNDING INFORMATION

None.

## CONFLICT OF INTEREST STATEMENT

None to declare.

## ETHICS STATEMENT

The manuscript has been reviewed and approved by the IRB and Public Affairs Office.

## CONSENT

The authors have confirmed that patient consent has been signed and collected in accordance with the journal's patient consent policy.

## Data Availability

The corresponding author can provide the datasets analyzed in this study upon request. Additionally, the manuscript appropriately cites the resources used for the review and is readily available.
